# Predictors and outcomes of engagement in an online depression prevention program for final year secondary school students

**DOI:** 10.1016/j.xjmad.2023.100027

**Published:** 2023-09-22

**Authors:** Hayley M. Jackson, Louise M. Farrer, Aliza Werner-Seidler, Yael Perry, Helen Christensen, Jeneva L. Ohan, Alison L. Calear, Philip J. Batterham

**Affiliations:** a Centre for Mental Health Research, National Centre for Epidemiology and Population Health, The Australian National University, Canberra, Australia; b Black Dog Institute, University of New South Wales, Sydney, Australia; c Telethon Kids Institute, The University of Western Australia, WA, Australia; d School of Psychological Science, The University of Western Australia, WA, Australia

**Keywords:** Engagement, Adherence, Adolescents, Depression, Internet interventions

## Abstract

Although school-based delivery of online interventions can effectively prevent depression and other common mental disorders, little is known about the characteristics of students who engage with these programs. This study aimed to identify predictors of two indicators of adolescent engagement (program usage and skill enactment) with a school-based online depression prevention program. The study also explored the association between skill enactment and mental health outcomes. Participants were 204 final-year secondary school students (*M* = 16.7 years, *SD* = 0.51 years; 111 females) from the intervention condition of a cluster randomized controlled trial conducted in 10 selective and partially selective government schools in Australia between 2015 and 2016. Program usage was measured as number of modules completed and skill enactment was assessed using a self-report questionnaire at post-intervention to determine the frequency of implementing program strategies (e.g., cognitive restructuring, activity scheduling). Predictors of engagement included demographics, mental health-related variables, and psychological factors (e.g., mastery). Regressions indicated that possible history of depression predicted fewer modules completed, whereas higher baseline anxiety, mastery, and higher perceived level of learning from the program predicted greater skill enactment. Mixed models did not show a significant effect of skill enactment on symptoms of depression, generalized anxiety, social phobia, or personal stigma. Future work is needed to investigate whether the results apply to students enrolled in non-selective programs and examine the effects of skill enactment and other possible mechanisms on outcomes in order to guide the refinement of future interventions.

## Highlights

• Limited research has explored engagement in school-based internet interventions.
• Different aspects of engagement were predicted by different factors.
• Mental health related variables and psychological factors predicted engagement.
• Use of strategies in daily life was not associated with improved outcomes.
• Future research should investigate the impact of engagement on outcomes.

## 1 Introduction

The implementation of digital mental health interventions (DMHIs) in schools is increasingly recognized as an effective way to prevent and treat depression and other common mental disorders among young people [1]. When delivered in schools, DMHIs preserve the benefits of school-based programs in terms of improved reach and accessibility while also providing key advantages over in-person delivery methods. These advantages include reduced demands on teaching staff who are not necessarily trained to support student mental health, decreased stigma associated with receiving mental health support, and high fidelity in intervention delivery [2]. However, challenges remain regarding how best to engage students with these programs. Relative to entirely self-guided approaches, school-based DMHIs have been associated with improved engagement among adolescents [3], [4]. Yet even in monitored settings, completion rates are often modest, and previous trials have indicated that only 30–60 % of adolescents fully complete these programs [5], [6], [7]. Given that engagement is a key factor in the effectiveness of DMHIs [8], [9], research that focuses on identifying the relationship between user characteristics and engagement may help guide the development of strategies to improve program effectiveness [10].

However, inherent in this line of research is a broader challenge regarding how to define and operationalize engagement with digital interventions. Typically, studies have used system usage data, such as the number of modules, sessions, or within-program activities completed, as a proxy for engagement [11]. Indicators of program usage can provide useful information about the rate of progress through an intervention or the depth of engagement with specific intervention components. Higher rates of program usage have also been associated with small to moderate improvements in mental health outcomes, irrespective of the type of intervention or symptoms targeted [8]. However, when collected in school settings, these indicators may depend not only on how motivated the student is but also on classroom and teacher characteristics [12]. In addition, they may not account for other behaviors that might be more proximal to outcomes, such as the application of knowledge and strategies learned from an intervention [9], [11].

Several studies have conceptualized the application of skills in everyday life (referred to as skill enactment hereafter) as an additional aspect of engagement in both digital and face-to-face interventions [13], [14], [15]. This definition extends the boundary of engagement research beyond technology usage to include the degree to which users engage with and apply intervention content in their daily lives. Evaluating skill enactment and its predictors may be especially relevant to research aimed at improving intervention effectiveness, given that it has been suggested to be more important for mental health outcomes than program usage measures [11], [16], is often only weakly correlated with technology usage [17], and may be a better indicator of active and meaningful engagement than automated usage data in school settings.

Relatively few studies of school-based DMHIs have attempted to identify user characteristics that are associated with either program usage or skill enactment indicators of engagement. Some studies have shown that greater program usage was predicted by living in a rural location, lower year level, higher pre-intervention depression, lower pre-intervention anxiety, and higher self-esteem [12], [18], [19], [20]. However, only one previous evaluation of an online depression program based on cognitive behavioral therapy (CBT) delivered in an educational setting has examined predictors of skill enactment among young people. This study found no evidence to suggest that skill enactment was associated with baseline symptoms [21]. Some studies have investigated predictors of skill enactment among adult users of DMHIs, but these have predominantly focused on the relationship between skill enactment and intervention-related factors (such as the presence of guidance) in the absence of considering other theoretically prescribed predictors of behavior [22]. Other factors that may be important for DMHI engagement, but which have been overlooked in school-based studies, include beliefs about the effectiveness of DMHIs, social influences, and in the case of skill enactment, the degree to which users interact with and acquire knowledge from an intervention program [14], [22], [23]. Thus, further research is needed to examine a more comprehensive array of predictors of both program usage and skill enactment indicators of engagement among users of school-based DMHIs.

The present study builds on previous studies of engagement with school-based DMHIs by investigating two indicators of engagement in the Trial for the Prevention of Depression (TriPoD). The TriPoD trial used a cluster randomized controlled trial (cRCT) design to test the effectiveness of a gamified online CBT intervention (SPARX-R) for the prevention of depression among final year secondary school students attending selective and partially selective government secondary schools in Sydney, New South Wales. In Australia, selective and partially selective schools use an entrance examination to admit students or place them in specialist streams (as is the case in partially selective schools) based on academic merit. The intervention was trialed in selective schools because, along with other known risk factors for mental health problems among young people (e.g., exposure to poverty or discrimination [24]), students attending high-achieving schools are increasingly recognized as a group at elevated risk for mental health problems [25]. Risk factors implicated in the development of mental health difficulties among this group include pressure to succeed from parents, schools, and peers, as well high levels of perfectionism and the competitive academic nature of the school environment [25], [26]. The intervention was trialed with students in their final year, as final course examinations represent a significant school-related stressor that may exacerbate these challenges and precipitate mental health problems [27].

The methods and primary outcomes of the trial have been published [7], [28], and indicated that the program was effective in reducing symptoms of depression relative to a control condition at post-intervention and 6-month follow-up. Furthermore, sub-group analyses showed that the intervention was effective in reducing depressive symptoms at post-intervention and 6 months for participants who completed 4 or more modules, but not for those who completed fewer modules [7]. Therefore, identifying factors associated with engagement may help guide the development of strategies to enhance effectiveness through improved program engagement. This study sought to extend on these initial findings and aimed to identify predictors of module completion and skill enactment in the active intervention condition. The study also aimed to explore the effect of skill enactment on primary (depression) and secondary (anxiety and personal depression stigma) outcomes.

## 2 Materials and methods

### 2.1 Overview

We performed a secondary analysis of data from the TriPoD trial, which used a cRCT design to test the effectiveness of an online CBT intervention (SPARX-R) for the prevention of depression among final year secondary school students relative to an attention-matched control condition. Assessments took place at baseline, post-intervention, and six- and 18-months after the baseline assessment. Full details on the methods and primary outcomes for the trial have been published elsewhere [7], [28]. The trial received ethics approval from the University of New South Wales (HC14105), the Australian National University Human Research Ethics Committee (protocol 2014/325), and the New South Wales Department of Education and Training (SERAP2014001).

### 2.2 Participants

Selective and partially selective government secondary schools from Sydney, New South Wales, Australia, participated in the trial between February 2015 and August 2016. All students at participating schools who were enrolled in their final year of high school (year 12) were invited to participate. For partially selective schools, enrollment status (selective or non-selective) was recorded. There were no exclusion criteria as the study tested a universal prevention program. Written informed consent was obtained from students and their parents prior to the start of the trial. Students who did not provide consent were offered access to the assigned intervention but did not complete research questionnaires.

### 2.3 Intervention: SPARX-R

The SPARX-R program was adapted from SPARX [29], a fully automated CBT-based program which teaches skills to adolescents experiencing mild to moderate symptoms of depression using an unguided, interactive fantasy game format. SPARX-R delivers the same content as SPARX but is framed in preventive terms. For example, whereas in the original program participants are informed that “SPARX was made to help young people who feel down or depressed”, SPARX-R informs participants that “this version of SPARX was made to help young people who are having hassles and feeling down, stressed or angry a lot of the time. Even if you are doing fine, SPARX-R can help strengthen your skills for dealing with problems when they do come along” [30]. The program is delivered across seven modules. Users navigate through a series of challenges and activities within a fantasy world that has been invaded by gloomy, negative automatic thoughts (GNATs). Their mission is to restore balance to the world. The challenges and activities are designed to help the user understand and apply CBT skills via play-based learning and the use of a virtual guide character who provides context and relates the content back to the user’s real-world experiences [30]. The modules and core skills focus on: (1) finding hope (negative automatic thoughts, controlled breathing), (2) being active (activity scheduling and behavioral activation, progressive muscle relaxation, communication and interpersonal skills), (3) dealing with strong emotions (assertiveness, listening, and negotiation), (4) overcoming problems (problem-solving, cognitive restructuring), (5) recognizing unhelpful thoughts (cognitive restructuring), (6) challenging unhelpful thoughts (cognitive restructuring, negotiation skills), and (7) bringing it all together (summary, mindfulness). Each module takes 20–30 min to complete. For more information on the original and revised versions of SPARX, see Merry et al. [29] and Fleming et al. [30].

Schools scheduled curriculum time for students to complete 1–2 modules per week over the course of 5–7 weeks, with supervision provided by classroom teachers. Research personnel were not directly involved in the delivery of the intervention.

### 2.4 Attention-matched control condition: lifeSTYLE

Participants assigned to the attention control condition received access to the online educational program, lifeSTYLE. This program is a modified version of an online program originally developed as a control intervention for a trial targeting reduction in suicidal thoughts [31]. The program was designed to match SPARX-R in terms of duration without providing skills to prevent or manage mental health difficulties. The program is delivered in seven modules (lasting 20–30 min), each of which provides knowledge and information on topics related to general health: (1) independence, (2) participating in your community, (3) work skills, (4) mobile phone safety and hygiene, (5) healthy skin, (6) sustainable eating and (7) maintaining a healthy home environment. Interactive activities are designed to reinforce knowledge and learning (e.g., quizzes, myth busters). Control group participants were excluded from this study.

### 2.5 Measures

#### 2.5.1 Engagement

Indicators of engagement included number of modules completed (ranging from 0 to 7) and frequency of enacting skills from the SPARX-R program. Module completion was automatically recorded within the program software, with modules marked as complete if the participant reached the end of the module. Skill enactment was assessed at post-intervention using a list of 13 strategies and techniques specific to the SPARX-R program. Respondents rated how often they had tried each of the skills on a 3-point scale (Never = 0, Sometimes = 1, Often = 2). Example items include: “Challenging GNATS (unhelpful thoughts)” and “Mindfulness (tolerating distress)”. These questions were based on items used in a previous trial of an online anxiety prevention program and adapted for SPARX [32]. Given the range of skills examined, exploratory factor analysis was conducted on the 13 skill enactment items. This analysis was consistent with the scale representing a single factor (see Supplementary material for further details). Therefore, we used the total scale (ranging from 0 to 26) as a measure of overall skill enactment, with higher scores indicating more frequent skill enactment. Cronbach’s alpha for the total scale at post-intervention was excellent (α = .92).

#### 2.5.2 Predictors of engagement

Predictors of engagement (i.e., program usage and skill enactment) included demographic variables, baseline mental health symptoms, and psychological factors. Each of the predictor variables described below were assessed at pre-intervention, except for perceived level of learning, which was assessed at post-intervention. Demographic variables were gender (female = 0, male = 1) and language spoken at home (English = 0, other = 1).

Mental health-related variables included depression symptoms measured by the Major Depression Inventory (MDI) [33] (α = .88 in the present sample) and the Generalized Anxiety (α = .80 in the present sample) and Social Phobia (α = .76 in the present sample) subscales of the Spence Children’s Anxiety Scale (SCAS) [34]. Subscale scores on the SCAS were summed to produce a total anxiety severity score (α = .85 in the present sample). The MDI has acceptable sensitivity and specificity for the diagnosis of depression according to ICD-10 and DSM-IV criteria [33], and the SCAS has been demonstrated to be a valid and reliable indicator of anxiety in adolescent samples [35]. Two standalone screening questions from the Structured Clinical Interview for DSM-IV disorders–Mood module were also included to provide an indication of possible history of depression [36]. The questions assess mood and anhedonia symptoms (α = .76 in the present sample), with respondents answering ‘yes’ or ‘no’ to whether they have ever experienced each symptom for two weeks or longer.

Psychological factors included personal depression stigma, sense of mastery, social support, confidence to learn skills, and perceived level of learning. Personal depression stigma was measured with the 9-item Personal Stigma subscale of the Depression Stigma Scale (DSS-Personal) [37], with each item rated on a 4-point scale from 0 (strongly disagree) to 4 (strongly agree). Total scores range from 0 to 36, with higher scores indicating greater stigma (α = .79 in the present study). Perceived mastery over life outcomes was measured with the 5-item version of the Pearlin Mastery Scale [38]. Each item is rated on a 4-point scale from 1 (strongly agree) to 4 (strongly disagree). Total scores range from 5 to 20, with higher ratings indicating a higher level of self-mastery (α = .75 in the present study). The 5-item scale was used in place of the original 7-item scale given evidence to support improved reliability and model fit for the shortened version [39], [40]. Positive social support from family and friends was measured using the supportive interactions subscales (4 items in total) from the Schuster Social Support Scale [41]. Each item was rated on a 4-point scale, from 0 (never) to 3 (often). Scores are computed separately for family (α = .81 in the present study) and friends (α = .71 in the present study), with item scores averaged to compute total scores.

Pre-intervention confidence to learn skills and perceived level of learning at post-intervention were assessed using single items that asked participants whether they were confident that people could learn skills to enhance wellbeing from an online program (rated on a 5-point scale, ranging from strongly disagree to strongly agree) and how much they perceived they had learnt from the program (rated on a 4-point scale, ranging from almost nothing to a great deal). These questions are also based on those used in previous research [32].

#### 2.5.3 Intervention outcomes

The primary outcome was symptoms of depression measured by the MDI. Secondary outcomes included symptoms of generalized anxiety disorder and social anxiety disorder measured using the SCAS and personal depression stigma measured by the DSS.

### 2.6 Analyses

Data were analysed using SPSS *v28*, and significant effects were determined at *p* < 0.05. Mixed effects ordinal logistic regression (GENLINMIXED command) was used to examine predictors of module completion, with a random intercept term included to account for the clustering of students within schools. Fixed effects linear regression analysis was used to identify predictors of skill enactment. A random effect for school was omitted from the regression on skill enactment, as the estimated intraclass correlation coefficient (ICC) and design effect (ICC = 0.009, design effect = 1.42) indicated the absence of a multilevel structure to the data. Some predictors were dichotomized for analysis: confidence to learn skills was re-coded into not confident (strongly disagree or disagree) and confident/neutral (strongly agree, agree or neither agree nor disagree), and perceived level of learning was re-coded into no/minimal learning (‘almost nothing’ or ‘not very much’) and some/considerable learning (‘a fair bit’ or a ‘great deal’).

Evidence for moderation of the primary and secondary outcomes by skill enactment was evaluated using mixed model repeated measures (MMRM) analysis of variance (ANOVA) with a two-way interaction term (i.e., skill enactment×time). The interaction term provides information on whether changes in outcome over time differed more for those who had higher levels of skill enactment. School was included as a random factor to reflect the clustered sampling of students within schools. An unstructured covariance matrix was used to model relationships between observations at different occasions, and error degrees of freedom were estimated using the Satterthwaite method.

## 3 Results

### 3.1 Participants

Table 1 presents the baseline characteristics and engagement data for the study sample. Module completion was both a predictor and outcome variable. Ten selective and partially selective government secondary schools participated in the trial during February 2015 and August 2016. A total of 540 final year secondary students (341 female, 199 male) with a mean age of 16.7 years (*SD* = 0.51) consented to participate and were randomized at the school-level to the intervention group (*n* = 242) or control group (*n* = 298). Of this group, the majority (*n* = 469, 54.4 %) returned post-intervention surveys, 341 (63.1 %) completed 6-month follow-up surveys, and 104 (19.3 %) completed 18-month follow-up. Sample size ranged from 7 to 126 participants from each school. Control group participants were excluded from the current analysis. After excluding a further 38 participants who did not have complete data on the included variables, data from a final sample of 204 participants were analysed (*n* = 4 students enrolled in non-selective programs). Chi-square analyses and *t*-tests demonstrated that there were no significant differences between those who did and did not have complete data on the basis of language spoken at home (*χ*^*2*^_1_ =.07, *p* = 0.796), confidence to learn skills (*χ*^*2*^_1_ *=*.18, *p* = 0.674), possible history of depression (*χ*^*2*^_1_ =.48, *p* = 0.490), pre-intervention depressive symptoms (*t*_240_ = −1.09, *p* = 0.276), anxiety (*t*_240_ = −1.00, *p* = 0.320), personal stigma (*t*_240_ = 0.11, *p* = 0.910), self-mastery (*t*_240_ = −1.44, *p* = 0.150), or supportive interactions with friends (*t*_240_ = 0.68, *p* = 0.500) or family (*t*_240_ = 1.16, *p* = 0.246). However, males (*χ*^*2*^_1_ = 3.96, *p* = 0.047) and participants who completed fewer modules (*t*_240_ = −5.47, *p* < 0.001) were more likely to have incomplete data.

**Table 1:** Baseline characteristics and engagement data for participants included in the study (*N* = 204).

	Intervention group (SPARX-R)
***Demographics***	
Gender, *n* (%)	
Male	93 (45.6)
Female	111 (54.4)
Language spoken at home, *n* (%)	
English	112 (54.9)
Other	92 (45.1)
***Mental health status***	
Depression severity (MDI), *M* (*SD*)	15.36 (8.23)
Anxiety severity (SCAS), *M* (*SD*)	13.97 (6.02)
Depression history, *n* (%)	48 (23.5)
***Psychological factors***	
Depression stigma (DSS-Personal), *M* (*SD*)	10.11 (5.29)
Mastery (PMS), *M* (*SD*)	14.22 (2.52)
Social support friends (SSS-friends), *M (SD)*	2.38 (0.57)
Social support family (SSS-family), *M (SD)*	2.50 (0.66)
Confidence to learn skills	
Not confident	32 (15.7)
Confident/neutral	172 (84.3)
***Post-intervention learning***	
Learning, *n* (%)	
Some/considerable learning	77 (37.7)
No/minimal learning	127 (62.3)
***Engagement***	
Modules completed, *M* (*SD*)	4.05 (2.13)
Skill enactment, *M* (*SD*)	9.64 (6.26)

### 3.2 Module completion and skill enactment

On average, participants completed approximately 4 program modules (*M* = 4.05, *SD* = 2.13), with a majority (106/204, 52.0 %) completing 4–6 modules and 26 (12.7 %) completing all 7 modules. However, almost one-third of participants (57/204, 27.9 %) completed only 1–3 modules and 15 participants (7.4 %) did not complete any modules. The mean for skill enactment was 9.64 (*SD* = 6.26), and the average number of skills tried (at least sometimes) was approximately 8 out of a total 13 (*M* = 7.69, *SD* = 4.31). One-fifth of participants (19.1%) reported trying all 13 skills, although 19 (9.3 %) had not practised any skills and a further 23 (11.3 %) practised only 1–3 skills. The most frequently practised skills included listening skills (*M* = 1.06, *SD* = 0.70; 78.4 % practised), activity scheduling (*M* = 0.94, *SD* = 0.68; 73.5% practised) and keeping busy (*M* = 0.85, *SD* = 0.68; 70.6 % practised). The least used skills were identifying positive and realistic thoughts (*M* = 0.47, *SD* = 0.64; 38.7 % practised), engaging in progressive muscle relaxation (*M* = 0.49, *SD* = 0.64; 40.7 % practised), and using problem-solving strategies (*M* = 0.55, *SD* = 0.68; 44.6 % practised).

### 3.3 Predictors of module completion

The mixed-effects ordinal logistic regression presented in Table 2 demonstrated that module completion was significantly related to possible history of depression, with possible history of depression being associated with a 54% decrease in the odds of completing an additional program module, after adjusting for the effects of other variables. There were no other significant predictors of module completion.

**Table 2:** Mixed-effects ordinal multinomial logistic regression model on number of modules completed (*N* = 204).

	Coefficient	*SE*	*OR*	95% CI	*p* value
Gender (Female)	0.51	0.56	1.67	0.55–5.09	0.364
Language (English)	-0.01	0.27	0.99	0.58–1.68	0.966
Depression	-0.02	0.02	0.98	0.94–1.02	0.319
Anxiety	0.00	0.03	1.00	0.95–1.06	0.965
Depression history	**-0.79**	**0.37**	**0.46**	**0.22–0.94**	0.033*
Depression stigma	-0.01	0.03	0.99	0.94–1.05	0.794
Perceived mastery	-0.11	0.06	0.90	0.80–1.01	0.066
Social support–friends	0.40	0.26	1.49	0.90–2.49	0.123
Social support–family	-0.21	0.22	0.81	0.52–1.25	0.342
Confidence to learn skills (not confident)	-0.25	0.35	0.78	0.39–1.55	0.480

*Note*:

* *p* < 0.05

### 3.4 Predictors of skill enactment

Table 3 presents the results of the linear regression on skill enactment. Greater levels of skill enactment were significantly associated with higher baseline anxiety, greater mastery, and greater perceived level of post-intervention learning. Perceived level of learning was the strongest predictor, with the model indicating that after controlling for all other factors, those who reported higher perceived learning from the program reported an average increase in skill enactment of 3.40 points, compared to those who reported learning little from the program*.* There were no other significant factors associated with skill enactment in the model.

**Table 3:** Linear regression model on skill enactment (*N* = 204).

	Coefficient	*SE*	*t*	95% CI	*p* value
Gender (Female)	-1.24	0.96	-1.29	-3.14–0.65	0.198
Language (English)	-1.33	0.87	-1.52	-3.05–0.39	0.129
Depression	-0.01	0.07	-0.18	-0.15–0.12	0.856
Anxiety	**0.21**	**0.09**	**2.39**	**0.04–0.38**	0.018*
Depression history	0.14	1.21	-0.12	-2.52–2.24	0.905
Depression stigma	-0.11	0.09	-1.16	-0.29–0.08	0.247
Perceived mastery	**0.57**	**0.20**	**2.90**	**0.18–0.96**	0.004**
Social support–friends	-0.31	0.83	-0.37	-1.96–1.33	0.710
Social support–family	0.41	0.73	0.56	-1.03–1.85	0.579
Confidence to learn skills (not confident)	-2.08	1.17	-1.78	-4.38–0.23	0.077
Modules completed	-0.17	0.22	-0.77	-0.59–0.26	0.445
Post-intervention learning (Some/considerable learning)	**3.40**	**0.96**	**3.55**	**1.51–5.28**	< 0.001***

*Note*:

* *p* < 0.05,

** *p* < 0.01,

*** *p* < 0.001.

### 3.5 Association between skill enactment and mental health outcomes

There were no significant overall interaction effects between post-intervention skill enactment and measurement occasion for any of the primary or secondary outcomes based on MMRM ANOVAs, including for depression (*F*_3108.59_ = 1.82, *p =* 0.148), generalized anxiety (*F*_3,88.43_ = 1.29, *p =* 0.282), social phobia (*F*_3,87.87_ = 1.31, *p =* 0.277), or personal depression stigma (*F*_3106.04_ = 2.44, *p =* 0.069). Contrasts demonstrated no significant interaction effects between time and skill enactment on symptoms of depression or anxiety at post-intervention and follow-up, although there was a significant interaction between time and skill enactment for depression stigma at post-intervention (*t*_202.00 *=*_ −2.62, *p* = 0.009, 95 % CI = −.26 to −.04), such that participants with higher levels of post-intervention skill enactment had greater reductions in depression stigma from baseline to post-intervention. The effect remains significant using a conservative alpha of *p* < 0.01 to account for multiple comparisons. The effect was not maintained at 6- (*t*_134.40 *=*_ −1.60, *p* = 0.111, 95 % CI = −.20 to.02) or 18-month (*t*_49.09 *=*_ −1.13, *p* = 0.264, 95% CI = −.25 to.07) follow-ups.

## 4 Discussion

This study built on previous research on adolescent engagement with DMHIs by investigating predictors of engagement, measured using modules completed and skill enactment, among a sample of final-year secondary school students participating in a school-based online depression prevention program. The study also explored whether skill enactment moderated the effect of the intervention on primary and secondary outcomes. The findings point to several considerations for future program design and delivery, as possible history of depression predicted fewer modules completed, whereas self-reported perceived level of learning from the program, higher levels of baseline mastery, and more severe baseline anxiety symptoms predicted skill enactment, suggesting that unique factors may underlie different aspects of adolescent engagement with school-based DMHIs. In addition, unlike evidence presented in the main trial paper, which showed a significant effect of module completion on reduction in depression symptoms, skill enactment was not shown to be related to reductions in either depressive or anxiety symptoms. However, despite a non-significant omnibus test, skill enactment was related to reductions in stigma at post-intervention.

The findings for module completion indicated that participants with a possible history of depression (i.e., those who reported prolonged experiences of mood or anhedonia symptoms) tended to complete fewer modules than those who did not. This finding warrants further investigation. Students with past depression have higher rates of school nonattendance than their peers [42], which may suggest that they had fewer opportunities to use the program during class time. However, it could also suggest that this group may not have found the program to be relevant to their situation or needs, perhaps because they had already received mental health support or because the content was focused on prevention rather than addressing their specific concerns. Personalized intervention content, and the availability of choice, has been suggested to be critical for motivation and engagement with DMHIs [43], [44]. Although this approach has potential to contribute to stigma when school-based programs are delivered in person [45], the relative confidentiality offered by self-directed online programs may allow for a greater degree of tailoring to student needs and preferences. We did not identify any other significant predictors of module completion. This contrasts with previous studies on adolescent engagement with online interventions in school-based and community settings, which have found baseline depression and levels of social support to predict program usage [18], [46]. The absence of findings related to these variables could be due to the delivery setting, as well as differences in how program usage was defined in this study as compared with previous studies (i.e., as modules, rather than activities, completed). Our decision to enter all predictors simultaneously into the predictive model to account for any possible confounding may also have contributed.

Unlike module completion, greater skill enactment was predicted by higher perceived learning from the program, higher levels of mastery, and more severe pre-intervention anxiety symptoms. The finding for perceived learning is broadly consistent with models of intervention engagement and behavior change [14], [47], both of which highlight having knowledge to perform a target behavior as an essential condition for actual behavioral enactment. This might provide an opportunity to improve skill enactment through the development of strategies to facilitate knowledge acquisition and memory for intervention materials. Recent studies have highlighted the potential of learning support strategies to improve engagement and outcomes [48], [49]. For example, Berg et al. [50] demonstrated a small but significant positive effect of learning support strategies (e.g., knowledge quizzes and pedagogical media such as pictures and video) on both knowledge acquisition and anxiety symptoms when integrated in an online CBT intervention for adolescent anxiety, relative to online CBT alone. However, the fact that we measured learning using participant self-reports could also suggest that participants who were more satisfied with the program, or perceived it to be more valuable, were more likely to try the skills compared with those who were less satisfied. To address this limitation, future studies should employ measures of declarative and procedural knowledge (e.g., multiple choice tests, reviews of homework, hypothetical scenarios) to confirm the findings reported here.

A greater sense of mastery was also associated with increased skill enactment, suggesting that young people who perceive an enhanced sense of agency over outcomes are more likely to implement program strategies. This finding supports research that has found sense of mastery to predict positive health behaviors and outcomes [51], and is consistent with evidence that a feeling of being in control of one’s health facilitates engagement with DMHIs [23]. As mastery captures a generalized sense of control over outcomes, future investigation of related control beliefs, such as task-specific self-efficacy to implement program strategies, may provide additional information on possible targets to facilitate skill enactment [52]. A range of strategies can be used to target self-efficacy beliefs, including enactive mastery, peer modeling, and social influence, and in the context of physical health, studies have demonstrated that self-management programs are associated with better outcomes when these strategies are incorporated [53]. In addition, the finding that more severe anxiety symptoms predicted greater skill enactment suggests that young people who were more anxious may have been more motivated or aware of their need for strategies to support their mental health or may have found the strategies provided in the program to be useful in addressing their symptoms, as treatment and prevention approaches for anxiety and depression can be similar.

Notably, there was no evidence that skill enactment was associated with number of modules completed, suggesting that usage of an online program delivered in schools is a poor indicator of what young people subsequently do offline. Universal programs face a challenge, as intervention recipients include both those who are experiencing symptoms of mental illness as well as many young people who are not currently experiencing mental health challenges. Consequently, factors such as the young person’s awareness of and need for mental health support are likely to influence whether program usage translates into skill enactment [45]. Furthermore, the fact that we identified few significant predictors of modules completed could suggest that factors related to the delivery setting (e.g., teacher and classroom characteristics) may be more important for engagement with mandatory program components than student characteristics in school-based DMHIs, consistent with Calear et al. [12]. As the trial on which this study is based demonstrated a positive association between module completion and improvement in depression symptoms [7], future research is needed to clarify how delivery setting impacts program usage. Moreover, focused research that explores student perspectives and predictors of non-participation among young people who choose not to participate in digital programs is needed to help improve engagement among this group, as they may face unique challenges to engaging with DMHIs. Examination of barriers to participation may also extend to parents, as many cases of non-participation in this study were due to non-return or refusal of parental consent.”

There was also no evidence to support a relationship between skill enactment and reductions in either depression or anxiety symptoms. This null finding is consistent with findings reported in a study targeting depression prevention among young people attending alternative education [54], but contrasts with evidence from treatment studies of DMHIs among adults [55], [56], [57], suggesting that the pattern of results described here may be related to the preventive nature of the program. Prevention studies often require significant long-term follow-up in order to demonstrate genuine prevention effects [1], and it may be that the length of the study period, paired with substantial loss to follow-up, was not sufficient to demonstrate benefits of skill enactment [45]. Alternatively, improving the frequency with which adaptive strategies are implemented may not be what helps produce symptom improvements in a prevention context. Instead, the program may have helped individuals to reduce their use of maladaptive strategies or improve the quality with which skills are enacted (i.e., how effectively the young person uses strategies in line with how the strategy was taught; [58]), such that ongoing use of specific adaptive strategies was not needed. The literature on emotion regulation and CBT skills use points to a range of possible mechanisms through which treatment strategies can reduce symptom severity [59], [60], and exploring these in future studies may help illuminate mechanisms of action in DMHIs targeting prevention. This research can help guide the targeted refinement and optimization of these interventions to be more efficacious and cost-effective. Finally, despite a non-significant overall finding for personal stigma, contrasts indicated an association between greater skill enactment and stigma reduction at post-intervention. This result should be regarded tentatively given the lack of an overall significant result, but it nevertheless warrants further investigation.

This study has several limitations. There are likely to be other engagement indicators not examined here that are also relevant for outcomes, such as the number of in-program activities completed or the extent to which students accessed additional supports [11]. Examining multiple aspects of engagement is essential to understanding the multifaceted nature of engagement with DMHIs. This may be aided in future trials by recent guidelines on collecting and reporting engagement data, such as those provided by Li et al. [11] and Beintner, Vollert [60]. In addition, our skill enactment measure was based on retrospective self-reports collected at post-intervention from the intervention group, meaning that participants may have over- or under-reported their use of strategies and we could not ascertain changes in skills from baseline to post-intervention or compare these changes against the control group. Collecting skill enactment data can be challenging as items measuring specific cognitive or behavioral strategies may not be readily understood by participants without previous exposure to CBT [61], but future trials should do so using measures that do not assume previous or current treatment engagement [61], [62]. Further, there are likely to be a range of other factors, including motivational (e.g., self-efficacy) and contextual factors (e.g., transitioning through final examinations and out of secondary education), which may have influenced the findings.

Our focus on high-achieving students enrolled in selective classes is also a limitation. Although this group of students may experience unique stressors that make them vulnerable to developing mental health difficulties [25], they may also be more self-motivated and proactive in their approach to engagement than students enrolled in non-selective programs. For example, some established predictors of academic success, such as individual differences in motivation, conscientiousness, and openness to experience [63], have also been shown to predict interest in and engagement with digital interventions [23]. Although no previous studies have investigated skill enactment following DMHIs among young people in mainstream schooling, our finding that more severe baseline anxiety symptoms predicted greater skill enactment contrasts with a previous study among youth in alternative education, which found no effect of initial symptom levels [21]. This might suggest that different factors may be important for DMHI engagement among different groups of young people. Consequently, future work is needed to understand how the findings described in this study apply to young people enrolled in mainstream and alternative education programs. Finally, our use of two screening questions from the Structured Clinical Interview for DSM-IV disorders–Mood module to provide an indication of past depression was not sufficient to establish a formal diagnosis of lifetime presence of depression. Future studies should include a comprehensive evaluation of past symptoms and functional impairment according to established classification systems (e.g., DSM-IV criteria).

## 5 Conclusions

This study identified several factors associated with engagement in a school-based depression prevention program among final-year secondary school students. These factors were differentially related to our engagement indicators. Adolescents with a possible history of depression tended to use the program less, whereas those with more severe baseline anxiety symptoms, higher levels of mastery, and those who reported learning more from the program enacted the skills they learned from the program more often. In addition, although module completion was shown to be related to improvement in the primary outcome of depression [7], there was no evidence to suggest that skill enactment moderated improvements in symptoms of depression or the secondary outcomes of anxiety and depression stigma. School-based prevention programs delivered online have considerable potential to reduce the prevalence and disease burden of depression and other common mental disorders, but limited understanding of what constitutes effective or meaningful engagement undermines our ability to design better interventions. Understanding processes of behavior change within DMHIs, including factors related to capability (e.g., learning processes, skill acquisition and practice) and motivation (e.g., self-efficacy beliefs and outcome expectancies), but also the dynamic environmental and social context within which engagement occurs, may provide a more rigorous and meaningful approach to studying engagement and guide the development of more appropriate services.

## Declaration of Competing Interest

The authors declare that they have no known competing financial interests or personal relationships that could have appeared to influence the work reported in this paper.
