# Supplementary material for: Predictors and outcomes of engagement in an online depression prevention program for final year secondary school students
Source: J Mood Anxiety Disord. 2023 Sep 22;3:100027. doi: 10.1016/j.xjmad.2023.100027 (PMC12244154; doi:10.1016/j.xjmad.2023.100027)
Supplement: Supplementary file 1 — Supplementary material [file mmc1.docx]

**Supplementary materials**

## Exploratory factor analysis

EFA with principal axis factoring was conducted to explore the factor structure of the skill enactment items. Sampling adequacy was verified by a Kaiser-Meyer-Olkin value of .901, and all diagonal elements on the anti-image correlation matrix exceeded .5 (range=.86–.95). Bartlett’s test of sphericity indicated adequate intercorrelations between items, *χ^2^*_78_=1478.32, *p* < 0.001. Based on parallel analysis (utilising principal component extraction with generation of 1,000 random data sets using the 95th percentile, and random permutations of the data set), one factor was retained accounting for 46.5% of the total variance. The construct was interpreted as representing overall skill enactment as it covered a broad range of skills relevant to the SPARX-R program. All item loadings exceeded 0.50, indicating adequate loadings on the factor (range = .505–.771) (Costello and Osborne, 2005). See Supplementary Table 1 for item-total correlations, communalities, and factor loading for the scale.

**Supplementary Table 1.** Item-total correlations, extracted communalities, and factor loadings for the 13-item skill enactment scale (*N*=204)

| ***Item*** | ***Item-total correlation*** | ***h^2^*** | ***Factor loadings*** |
| --- | --- | --- | --- |
| Negotiating | 0.734 | 0.594 | 0.771 |
| Challenging GNATS | 0.720 | 0.587 | 0.766 |
| Problem solving using STEPS | 0.715 | 0.574 | 0.757 |
| Mindfulness | 0.718 | 0.567 | 0.753 |
| Identifying SPARX thoughts | 0.709 | 0.559 | 0.748 |
| Recognising GNATS | 0.688 | 0.539 | 0.734 |
| Listening | 0.705 | 0.537 | 0.733 |
| Being assertive not aggressive | 0.676 | 0.498 | 0.706 |
| Keeping busy | 0.589 | 0.375 | 0.613 |
| Planning activities that give you a sense of pleasure and/or mastery | 0.575 | 0.355 | 0.595 |
| Progressive muscle relaxation | 0.568 | 0.345 | 0.587 |
| Asking for help | 0.497 | 0.262 | 0.511 |
| Controlled breathing | 0.488 | 0.255 | 0.505 |
|  |  |  |  |
| Eigenvalue |  |  | 6.05 |

*Note*: *h^2^* denotes communalities.
